# Islington War Memorial

**Published:** 1919-06-28

**Authors:** Gilbert A. Panter

**Affiliations:** Hon. Sec. Borough of Islington War Memorial Fund. Office: 566 Holloway Road, N. 7.


*To the Editor of* The Hospital.

### Islington War Memorial.

*To the Editor of* The Hospital.

Sir,—One of the first steps to be taken by the Borough of Islington War Memorial Committee will be to form a complete list of Islingtonians of the Naval, Military, Air, and Auxiliary Forces (men and women) who have fallen in the Great War. The names are to be inscribed in the public entrance hall of the proposed Memorial Extension of the Great Northern Central Hospital.

The Committee will be glad to receive from your readers the name, rank, and regiment or auxiliary service of any friend or relative previously resident in Islington with particulars of former address.

I am, Sir,

Yours obediently,

Gilbert A. Panter, Hon. Sec.

Borough of Islington War Memorial Fund.

Office: 566 Holloway Road, N. 7.

